# Comprehensive analysis of systemic, metabolic, and molecular changes following prospective change to low-carbohydrate diet in adults with type 2 diabetes mellitus in India

**DOI:** 10.3389/fnut.2024.1394298

**Published:** 2024-08-30

**Authors:** Nikhil Suresh Bhandarkar, K. Bhujang Shetty, Naren Shetty, Keerthy Shetty, Anupama Kiran, Narendra Pindipapanahalli, Rohit Shetty, Arkasubhra Ghosh

**Affiliations:** ^1^GROW Research Laboratory, Narayana Nethralaya Foundation, Bangalore, India; ^2^Narayana Nethralaya, Bangalore, India

**Keywords:** type 2 diabetes mellitus, low-carb diet, hyperglycemia, tear fluid analysis, biomarker discovery

## Abstract

**Purpose:**

South Asians, especially Indians, face higher diabetes-related risks despite lower body mass index (BMI) compared with the White population. Limited research connects low-carbohydrate high-fat (LCHF)/ketogenic diets to metabolic changes in this group. Systematic studies are needed to assess the long-term effects of the diet, such as ocular health.

**Method:**

In this prospective, observational study, 465 candidates aged 25–75 years with type 2 diabetes included with institutional ethics approval. A total of 119 subjects were included in the final study assessment based on the availability of pathophysiological reports, tears, and blood samples collected at baseline, 3rd, and 6th months. Serum and tear samples were analyzed by an enzyme-linked lectinsorbent assay, to examine secreted soluble protein biomarkers, such as IL-1β (interleukin 1 Beta), IL-6 (interleukin 6), IL-10 (interleukin 10), IL-17A (interleukin 17A), MMP-9 (matrix metalloproteinase 9), ICAM-1 (intercellular adhesion molecule 1), VEGF-A (vascular endothelial growth factor A), and TNF-α (tumor necrosis factor-alpha). A Wilcoxon test was performed for paired samples. Spearman’s correlation was applied to test the strength and direction of the association between tear biomarkers and HbA1c. *p*-value of < 0.05 was considered significant.

**Results:**

After a 3- and 6-month LCHF intervention, fasting blood sugar decreased by 10% (Δ: −14 mg/dL; *p* < 0.0001) and 7% (Δ: −8 mg/dL; *p* < 0.0001), respectively. Glycated hemoglobin A1c levels decreased by 13% (Δ: −1%; *p* < 0.0001) and 9% (Δ: −0.6%; *p* < 0.0001). Triglycerides reduced by 22% (Δ: −27 mg/dL; *p* < 0.0001) and 14% (Δ: −19 mg/dL; *p* < 0.0001). Total cholesterol reduced by 5.4% (Δ: −10.5 mg/dL; *p* < 0.003) and 4% (Δ: −7 mg/dL; *p* < 0.03), while low-density lipoprotein decreased by 10% (Δ: −11.5 mg/dL; *p* < 0.003) and 9% (Δ: −11 mg/dL; *p* < 0.002). High-density lipoprotein increased by 11% (Δ: 5 mg/dL; *p* < 0.0001) and 17% (Δ: 8 mg/dL; *p* < 0.0001). At the first follow-up, tear proteins such as ICAM-1, IL-17A, and TNF-α decreased by 30% (Δ: −2,739 pg/mL; *p* < 0.01), 22% (Δ: −4.5 pg/mL; *p* < 0.02), and 34% (Δ: −0.9 pg/mL; *p* < 0.002), respectively. At the second follow-up, IL-1β and TNF-α reduced by 41% (Δ: −2.4 pg/mL; *p* < 0.05) and 34% (Δ: −0.67 pg/mL; *p* < 0.02). Spearman’s correlation between HbA1c and tear analytes was not statistically significant.

**Conclusion:**

The LCHF diet reduces the risk of hyperglycemia and dyslipidemia. Changes in tear fluid protein profiles were observed, but identifying promising candidate biomarkers requires validation in a larger cohort.

## Introduction

Type-2 diabetes mellitus (T2DM) is one of the most prevalent chronic metabolic diseases. Approximately 537 million adults (20–79 years) are diagnosed with diabetes, and it is estimated that it will increase to 643 million by 2030 and 783 million by 2045. For approximately 116 million diabetic patients, China has the highest contribution. India ranks second with 77 million people, followed by the United States of America with 31 million, indicating that India is one of the most diabetes-risk countries in the coming decades (1, 2). Chronic diabetes-related complications are also a significant risk factor for cardiovascular diseases, chronic renal failure, diabetic retinopathy, and several other allied comorbidities (3). The economic burden of T2DM for treatment and management of its complications contributes to approximately 12% of global health expenditure (2).

Several ocular complications include T2DM, for instance, cataract, glaucoma, retinopathy, punctuate keratitis, and recurrent corneal lesions (4, 5). Patients with diabetes are known to experience dry eye as a common ocular symptom. Dry eye disease (DED) is considerably more common in diabetics than in healthy individuals, and it is also more common in those with T2DM than in those with type 1 diabetes mellitus (T1DM) (6, 7). T2DM is the primary cause of blindness in industrialized countries for individuals aged 25–74 years (8), which is the fourth reason of blindness in developing countries (9). Asia comprises both of the top two nations with the highest number of DM patients, namely, China (116 million) and India (77 million) (10), revealing the decade-long fast economic growth and urbanization in Asia, together with notable dietary and lifestyle changes (11, 12).

Genetic and sedentary lifestyles are crucial factors, especially diet, which play a significant role in the pathogenesis of T2DM. Lifestyle factors are reversible and focused on the effort to lower the T2DM-associated risk (13). Several systematic reviews and meta-analyses (SRMA) of randomized controlled trials (RCT) (14–16) revealed that interventions such as modified diet and/or enhanced level of physical activity can reverse or prevent the onset of T2DM and significantly contribute to the effective management of the disease (7). In RCT and observational studies, low-carbohydrate, high-fat (LCHF) diet or ketogenic diet has revealed promising improvements in glycemic control and weight loss, along with decreases in the quantity and/or doses of anti-diabetic medications (17–19). Low-carbohydrate approaches are supported mainly by the hypothesis that reducing insulin secretion, a critical hormone that creates an anabolic, fat-accumulation state, results in improved cardiometabolic function and induces weight loss (20). This approach has been recently called the carbohydrate–insulin model (21).

People of Indian origin or South Asian origin have a distinct pathophysiological feature with a higher risk of cardiometabolic disorders compared with the White population even though they have lower body mass index (BMI). This phenomenon is known as the “South Asian Phenotype” (21, 22). Distinct features such as abdominal adiposity combined with glucose intolerance and dyslipidemia such as high levels of triglycerides (TGs) and low-density lipoprotein (LDL), low levels of high-density lipoprotein cholesterol (HDL), and high levels of TGs relative to HDL with normal BMI (21–23). Studies have also revealed additional features, such as excess body fat per unit BMI, truncal obesity, higher c-reactive protein, and lower adiponectin as part of the South Asian phenotype (23, 24). Therefore, a myriad of different diabetes-control diets has gained significant popularity in India, including LCHF diets; however, biochemical or molecular data regarding the performance of such diets have not been adequately described.

Even with established ethnic variations in South Asian body composition and metabolic markers, there is a lack of studies connecting low-carbohydrate or ketogenic diets to metabolic alterations in the diabetic population in India and South Asia. Moreover, to understand and document, ocular changes after a switch over to ketogenic diets, though anecdotally described, require systematic long-term prospective trials. The ketogenic diet or low carbohydrate high fat (LCHF) diet-induced metabolic flexibility, adaptive response of an organism’s metabolism to maintain energy homeostasis to utilizing available fuels in situations in the shortage of carbohydrates as fuel, allowing the body to switch from using glucose as the chief fuel to using ketones instead, which has been previously documented in starvation as well as ketogenic diets in cases of epilepsy (25–27), causes a reduction in blood insulin, a rise in glucagon and depletion of visceral as well as subcutaneous fat stores and subsequently, leads to recovery in glycaemic factors. We aim to correlate this proposed metabolic reprogramming with changes in body mass index (BMI), body fat measurements, and metabolic parameters in subjects with T2DM. We further hypothesize that the hyperglycemia-induced elevated inflammatory molecular factors will be reduced by the LCHF diet. Such a reduction in the soluble factors in tear and serum may help understand the role of diabetes-associated inflammation in the progression of peripheral organ-specific complications, such as diabetic retinopathy (DR).

## Methods and materials

### Study design

This was an observational, prospective study performed after it was approved by the Narayana Nethralaya Institutional Ethics Committee (EC reference No: NNIEC/2022/02/01). A written consent form for the study was obtained from the subjects for their participation. For this study, samples from subjects with T2DM adopting the LCHF diet and those receiving standard care were collected. All clinical and disease progression histories were noted before (baseline) and after initiation at 3 and 6 months of home dietary intervention. Subjects with diabetes presenting to the retina clinic were also enrolled in the study (400–600). A total of 465 diabetic subjects were interviewed through counseling and guidance. The subjects with T2DM were treated for their condition as per the standard care with the addition of the LCHF diet. Participants were closely monitored, and their past and current treatment histories were noted in this study. A total of 119 participants and sex-matched diabetic subjects under standard care were finalized and recruited but not on the diet as controls. The inclusion criteria for participants were: person with T2DM, age 18–75 years, and HbA1c > 6.5%, while the exclusion criteria were person with T1DM and had serious diseases such as CKD, IHD, cancers, and DR, and wetting length of <8 mm.

### Dietary instruction for the LCHF diet

On recruitment, subjects provided a food log or a list of regularly consumed foods, such as drinks and snacks, to the nutritionist who educated them on carbohydrate metabolism and the role of insulin in lipogenesis and weight gain in simplified terminology. Subjects were recommended to restrict net carbohydrate (total carbohydrates minus fiber) intake to ≤50 g/day or 10–20% of their total calories, which is lower than the guideline of the LCHF diet. The daily recommendation for protein was capped at 20–25% of total calories based on their sex, physical activity level, and ideal body weight. The recommended total fat intake was 65–70% of total calories. Permitted food and beverages include meats, poultry, fish, eggs, low-carbohydrate nuts, seeds, non-starchy (over ground) vegetables, high-fat dairy products, fats, and oils such as olive oil, butter, and coconut oil, and beverages such as water and unsweetened tea or coffee. Sample meals, snack options, and recipes available online were discussed. Subjects were advised to eat only when hungry and avoid eating late at night. No caloric restriction was imposed. All subjects were recommended to drink eight glasses of water per day and encouraged to keep a food log. Food logs were reviewed at subsequent visits to monitor diet adherence.

### Clinical examinations

Data were collected longitudinally (0, 3, and 6 months) as part of the follow-up to determine the health status. A general exam was conducted at each visit to document the height, weight, blood pressure (BP), waist, and hip circumference. Following this, a detailed, whole blood serum analysis was performed at each visit.

### Sample collections

Blood and tear samples were collected at each visit according to the protocol. Tears were collected and processed according to the standard protocols of the clinical follow-up regime of the institute based on their disease status.

In brief, tear fluid was collected with the help of sterile Schirmer’s strips (5 × 35 mm^2^; Contacare Ophthalmics and Diagnostics, Gujarat, India) on each visit. The strips were positioned one in each of the conjunctival fornices, simultaneously in both eyes. The strips were collected after a sufficient amount of tear fluid (wetting of strips until the 20 mm mark or more) was absorbed and stored in a sterile microcentrifuge tube at −80°C. For extraction of tear fluid, Schirmer’s strips were chopped into small pieces; 300 μL of 1x sterile PBS was added and agitated for 2 h at 4°C at 300 rpm and centrifuged to elute the tear fluid. The elute was collected as tear fluid and used for further processing to examine secreted soluble protein factors in tears as described previously (28).

### Blood investigations

Blood samples were collected according to the standard protocol at each visit to monitor the metabolic changes. The following tests were performed: fasting blood sugars (FBS), post-prandial blood sugars (PPBS), glycosylated hemoglobin (HbA1C), renal function tests (RFT), liver function tests (LFT), lipid profile, serum insulin, C peptide, erythrocyte sedimentation rate (ESR), C reactive proteins (CRP), and blood ketones.

### Assessment of the diabetic medication effect score (MES)

The medication effect score (MES) was assessed by types (category by drug mechanism) and numbers (total number of oral hypoglycemic agents and injected insulins) at each visit (29).

The MES was the overall consumption of anti-glycemic agents. It was calculated by the sum of the median absolute decline of HbA1c times the percentage of the maximum daily dose for each medication, including insulin (30, 31). The higher the MES shown, the greater the utilization of medication. The assessment was performed at the beginning and the end of the 3-month RCT study.

### Tear evaluations

Tear samples were collected using Schirmer’s strips according to the standard clinical protocols, which required 2–4 min. The wetting length was noted, and the strips were immediately taken for testing or stored at −80°C in Eppendorf tubes for further evaluation.

### Multiplex ELISA

Using a variant of the multiplex ELISA assay (Biomarker Pathfinder, Novomol-Dx, India and Bio-Techne, USA), IL-1β (interleukin 1 beta), IL-6 (interleukin 6), IL-10 (interleukin 10), IL-17A (interleukin 17A), MMP-9 (matrix metalloproteinase 9), ICAM-1 (intercellular adhesion molecule 1), VEGF-A (vascular endothelial growth factor A), and TNF-α (tumor necrosis factor-alpha) were measured. Schirmer’s strips were collected in 1.5 mL tubes, in which 300 μL of phosphate buffer solution (extraction buffer) was added. The cartridge was loaded into an analyzer system, which indicated the measured value based on established internal references for each analyte. In the case of blood samples, serum was prepared for each respective sample. In total, 50 μL of serum was used in the same multiplex ELISA, to test changes in systemic levels of these analytes.

### Follow-up

Data of anthropogenic factors and blood and tear samples were collected longitudinally (0, 3, and 6 months) as part of the follow-up, to determine the disease and the ocular status.

### Statistical analyses

The Shapiro–Wilk test was applied to determine the distribution of the data. As the data were not normally distributed, Friedman test with Dunn’s multiple comparison test was performed to determine the significant difference in the parameters between baseline and 3- and 6-month follow-up visits and differences or inter-eye correlation, if any, in the tear fluid levels of analytes between the eyes, at all visits. To study the systemic effect of diet on overall ocular conditions, the average value of the analytes of tear from two eyes of the same subject was used. Since the data were not normally distributed, the repeated measure analysis by mixed-effect model-based analysis of variance (ANOVA) was not applicable. Furthermore, Spearman’s rank correlation coefficient was performed to determine the association between the various parameters studied. There was no statistical difference between the values of the two eyes in the cohort. All data are presented as median (interquartile range, IQR) distributed variables. A two-tailed *p*-value of ≤0.05 was considered statistically significant. Statistical analysis was performed using GraphPad Prism v.8 and MedCalc MedCalc^®^ Statistical Software version 20.218 (MedCalc Software Ltd., Ostend, Belgium).

## Results

### Enrollment and participant characteristics

**Figure 1:**
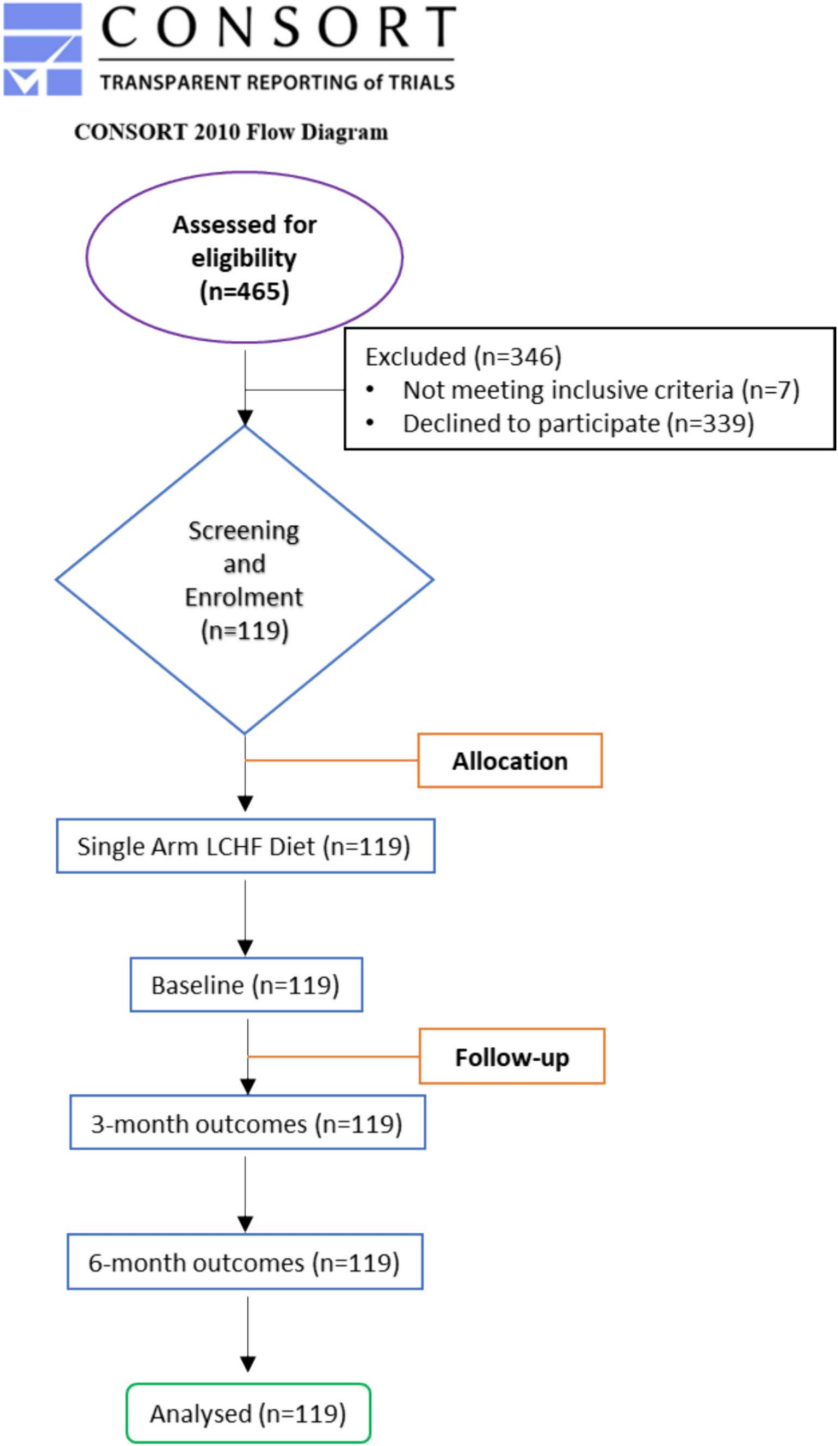
CONSORT diagram. Flow diagram of the study through the two time-points (enrolment, intervention allocation, follow-up, and data analysis). Adapted from Schulz et al. (32) licensed under CC BY-NC 2.0.

**Figure 2:**
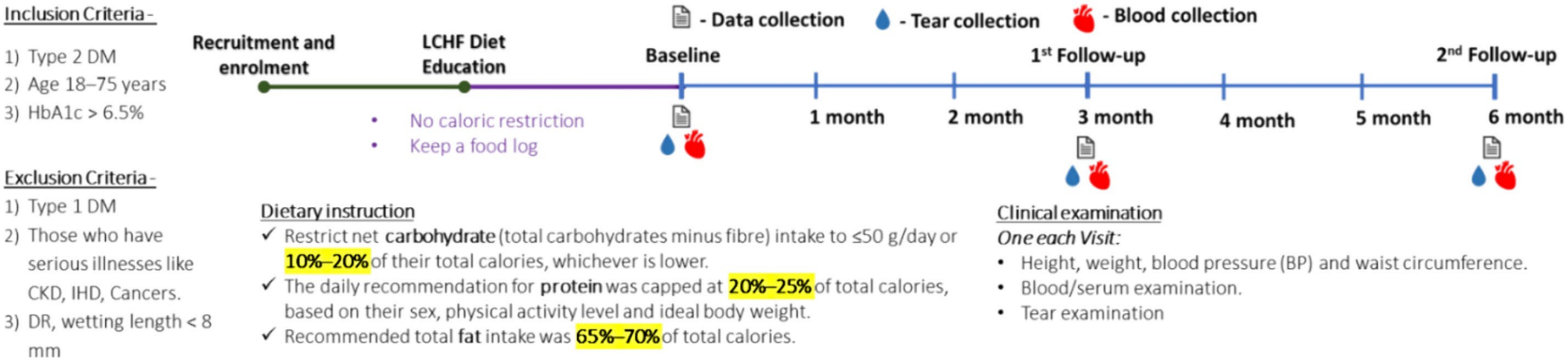
Experimental design. Schematic representation of the observational study design.

Out of the 465 enrolled participants, 7 dropped out as they did not meet inclusive criteria, and 339 candidates declined to participate in the study. Thus, 119 participants completed 3- and 6-month studies. The flow of the study is presented in Figure 1. In no instance was the primary reason for subject withdrawal attributed to the LCHF study. There were also no adverse events associated with the adoption or maintenance of the LCHF study (Figure 2).

### Anthropogenic factors

The results of anthropogenic parameters are presented in Table 1. All participants lost body weight and BMI at 1st and 2nd follow-up (Figure 3). On average, weight decreased by 1.3% for 1st follow-up (Δ: −1 kg; *p* < 0.00001) and by 2.2% at 2nd follow-up (Δ: −1.6 kg; *p* < 0.00001), while BMI decreased by 1.2% at 1st follow-up (Δ: −0.29 kg/m^2^, *p* < 0.00014) and by 1.8% at 2nd follow-up (Δ: −0.46 kg/m^2^; *p* < 0.00014) compared with their respective baseline (Table 1).

**Table 1:** Baseline and follow-up characteristics of the participants and changes in anthropometric and biochemistry parameters after LCHF diet intervention^#^.

Variable	BL^1^			F1^2^			F2^3^			Friedman test (Paired sample)		
	*N*	Median	IQR	*N*	Median	IQR	*N*	Median	IQR	*N*	*p*-value^a^	Multiple comparisons
**Anthropogenic**												
Gender (male/ female)	119	75/44										
Age (years)	119	52	42.3–60									
Weight (kg)	116	68.3	62.4–78	118	67.9	61.1–76.1	119	68	60.05–75.5	115	0.00001	(1) vs. (2); (1) vs. (3)
BMI	116	25.4	23.35–28.12	117	24.97	22.87–27.92	118	24.925	22.79–27.75	114	0.00014	(1) vs. (2); (1) vs. (3)
Waist (cms)	108	95	88.75–103.25	116	94	87–99.5	119	93	86–97.88	106	<0.00001	(1) vs. (2); (1) vs. (3)
HIP (cms)	108	100	96–104.25	116	99	95–105	119	99	93.25–104	106	0.00001	(1) vs. (3)
WHR	108	0.94	0.89–1	116	0.92	0.88–0.98	119	0.93	0.88–0.99	106	0.01113	(1) vs. (2)
**Glycaemic factors**												
FBS (mg/dL)	119	144	109–191.75	119	129	104–149.75	119	133	109.25–163	119	0.00002	(1) vs. (2); (1) vs. (3)
PPBS (mg/dL)	119	227	156.25–278.75	119	165	124.25–214.75	119	160	130.25–238.75	119	<0.00001	(1) vs. (2); (1) vs. (3)
Serum creatinine (mg/dL)	119	1	0.9–1.1	119	1	0.9–1.1	119	0.9	0.8–1.09	119	0.00102	(1) vs. (3)
SBP (mmHg)	119	130	120–150	119	130	120–140	119	120	120–130	119	<0.00001	(1) vs. (2); (1) vs. (3)
DSP (mmHg)	119	80	80–90	119	80	80–87.5	119	80	80–90	119	0.42064	–
Urea (mg/dL)	119	23	18–28	118	28	22–37	119	28	22–33.75	118	<0.00001	(1) vs. (2); (1) vs. (3)
Serum Insulin (Fasting) (mu/l)	113	10.24	6.88–14.09	109	11.94	6.97–14.7	114	9.65	6.32–13.38	98	0.46745	–
C Peptide (Fasting)	91	1.84	1.31–2.69	100	2.49	1.92–3.25	113	2.62	1.95–3.61	78	<0.00001	(1) vs. (2); (1) vs. (3)
Serum Ketone (mmol/L)	88	0.1	0.1–0.2	108	0.1	0.1–0.2	118	0.1	0.1–0.1	88	0.00079	(1) vs. (3)
QUICKI	113	0.32	0.3–0.34	109	0.32	0.3–0.34	114	0.32	0.31–0.34	98	0.24233	–
HOMA-IR	113	3.4	2.25–5.61	109	3.5	1.98–4.76	114	3.21	2.23–4.34	98	0.27493	–
HBA1C (%)	119	8.7	7.4–10.2	119	7.3	6.7–8.28	119	7.6	6.8–8.88	119	0.00003	(1) vs. (2); (1) vs. (3)
**Lipid profile**												
Total cholesterol (mg/dL)	119	195	162–232	118	173.5	143–216	119	185	154–216	118	0.0436	(1) vs. (2)
Triglycerides (mg/dL)	119	136	94.25–190.75	118	102	77–129	119	112	82–151	118	<0.00001	(1) vs. (2); (1) vs. (3)
HDL (mg/dL)	119	45	39.25–52	118	50	43–59	119	54	47.25–61.75	118	<0.00001	(1) vs. (2); (1) vs. (3)
LDL (mg/dL)	119	123	85.25–154	118	99.5	71–131	119	106	78–135.75	118	0.02485	(1) vs. (2); (1) vs. (3)
VLDL (mg/dL)	119	26	19–38	118	21	15–27	119	22	16–30	118	<0.00001	(1) vs. (2); (1) vs. (3)
Urine micro albumin (mg/L)	119	12	6.85–26.25	118	10	5–23	119	10	6–19	118	0.00987	(1) vs. (2); (1) vs. (3)
Uric Acid (mg/dL)	119	4.9	4.15–5.9	118	5	4–5.9	119	4.9	4.1–5.9	118	0.28483	–
**Liver Function test**												
Total Bilirubin (mg/dL)	119	0.7	0.5–0.9	118	0.7	0.5–0.9	119	0.7	0.6–1	118	0.00077	(1) vs. (3)
Direct Bilirubin (mg/dL)	119	0.2	0.2–0.3	118	0.2	0.2–0.3	119	0.2	0.2–0.3	118	0.40908	–
SGOT (U/L)	119	20	17–26	118	20	16–23	119	18	15–22	118	0.00778	(1) vs. (2); (1) vs. (3)
SGPT (U/L)	119	24	18–33.68	118	21.65	17–29.9	119	20.8	15.55–29.23	118	0.0004	(1) vs. (2); (1) vs. (3)
Alkaline phosphatase (U/L)	119	145	100–197	118	76	61–102	119	74	60.25–91	118	<0.00001	(1) vs. (2); (1) vs. (3)
**Thyroid profile**												
T3 (ng/mL)	119	1	0.9–1.2	118	0.9	0.8–1	119	0.9	0.8–1	118	0.00114	(1) vs. (2); (1) vs. (3)
T4 (ng/mL)	119	8.5	7.2–10.18	118	8.25	6.9–9.1	119	7.9	6.7–9.18	118	0.00037	(1) vs. (2); (1) vs. (3)
TSH (ng/mL)	119	2.2	1.33–3.68	118	2	1.3–3.2	119	1.9	1.2–3.28	118	0.006	(1) vs. (2); (1) vs. (3)
**Complete blood count**												
Hemoglobin (g/dL)	119	14.1	13–15.3	118	14.15	13.1–15.3	119	14.2	12.9–15.08	118	0.4871	–
WBC count (cell/cumm)	119	7,200	6,400–8,500	118	7,250	6,500–8,400	119	6,900	6,125–8,100	118	0.03982	(1) vs. (3)
Platelet (lakhs/cumm)	119	2.63	2.29–3.11	118	2.575	2.17–2.99	119	2.49	2.09–2.98	118	0.00003	(1) vs. (2); (1) vs. (3)
ESR (mm/h)	119	13	5–24	118	11	5–20	119	12	5–20.75	118	0.02142	(1) vs. (2); (1) vs. (3)
CRP (mg/L)	119	4.5	4.5–4.5	118	4.5	4.5–4.5	119	4.5	4.5–4.5	118	0.29443	–
**Serum electrolytes**												
Na + (mmol/L)	119	141.5	139.4–143	118	141.3	140.1–142.5	119	140.8	139.9–141.7	118	0.02467	(2) vs. (3)
K+ (mmol/L)	119	4.23	4.06–4.51	118	4.265	4.08–4.47	119	4.19	4–4.39	118	0.28365	–
Cl- (mmol/L)	119	101.8	99.85–103.4	118	103.4	101.4–105.4	119	104.6	103.13–105.9	118	<0.00001	(1) vs. (2); (1) vs. (3)
**Medication score**												
MEDS	99	1.317	0.9–1.89	79	1.317	0.96–1.61	34	1.25	0.65–1.9	31	0.02749	(1) vs. (2)

# Data presented in median (IQR); *p < 0.05, **p < 0.01, ***p < 0.001, and ****p < 0.0001, ^a^Friedman test (paired samples). ^1^BL – Baseline, ^2^F1 – 1st follow-up at 3 months from baseline, ^3^F2 – 2nd follow-up at 6 months from baseline; (1) vs. (2) – BL vs. F1; (1) vs. (3) – BL vs. F2; BMI, Body Mass Index; WHR, Waist Hip ration; FBS, Fasting Blood Sugar; PPBS, Post-Prandial Blood Sugar; SBP, Systolic Blood Pressure; DBP, Diastolic Blood Pressure; QUICKI, Quantitative Insulin Sensitivity Check Index; HOMA-IR, Homeostatic Model Assessment for Insulin Resistance; HBA1C, Glycated Hemoglobin; HDL, High-Density Lipoprotein; LDL, Low-Density Lipoprotein; VLDL, Very Low-Density Lipoprotein; SGOT, Serum Glutamic-Oxaloacetic Transaminase; SGPT, Serum Glutamic Pyruvic Transaminase; T3, Triiodothyronine; T4, Tetraiodothyronine; TSH, Thyroid Stimulating Hormone; WBC, White Blood Cell; ESR, Erythrocyte Sedimentation Rate; CRP, C-Reactive Protein; MES, Medication Effect Score.

**Figure 3:**
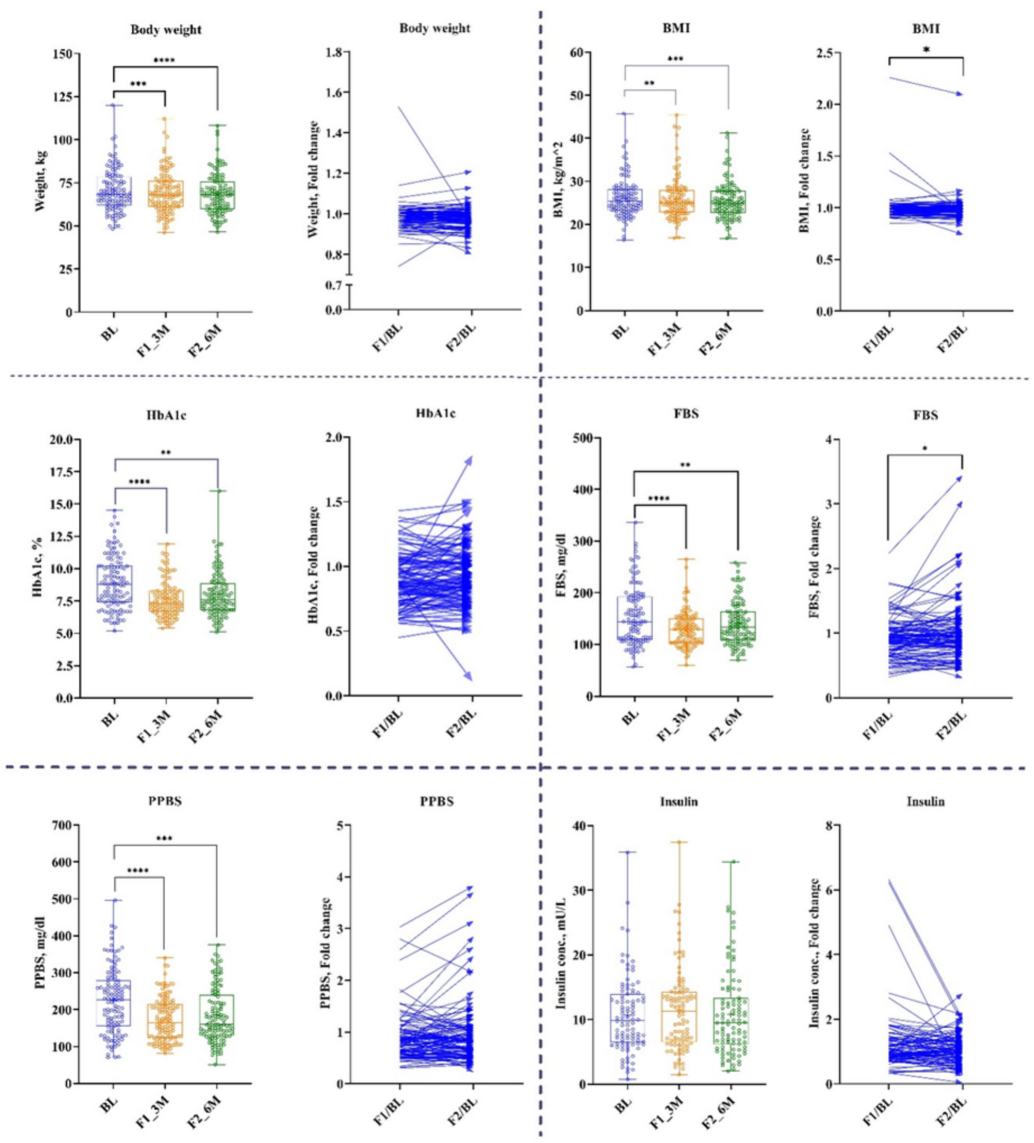
Anthropogenic and Glycemic parameters. Baseline – BL; 1st follow-up at 3 months – F1_3M; and 2nd follow-up at 6 months – F2_6M. Data points for body weight, BMI, HbA1c, fasting blood sugar (FBS), post-prandial blood sugar (PPBS), and insulin presented in median (IQR). *N* = 119. *p*-value of <0.05 is considered significant. *p < 0.05, **p < 0.01, ***p < 0.001, and ****p < 0.0001.

### Glycemic factors

Participants showed a significant reduction in FBS and PPBS at both time-points. FBS was decreased at 1st follow-up by 10% (Δ: −14 mg/dL; *p* < 0.00002) and at 2nd follow-up by 7% (Δ: −8 mg/dL; *p* < 0.00002) from baseline. Similarly, PPBS decreased by 23% (Δ: −42 mg/dL; *p* < 0.00001) and 20% (Δ: −43 mg/dL; *p* < 0.00001) at 1st and 2nd follow-ups, respectively from baseline. Correspondingly, HbA1c reduced by 12% (Δ: −1%; *p* < 0.00003) and 9% (Δ: 0.6%; *p* < 0.00003) at 1st and 2nd follow-ups, respectively, from baseline (Figure 3 and Table 1).

Nevertheless, fasting insulin and insulin resistance/sensitivity parameters such as homoeostasis model assessment (HOMA-IR) and quantitative insulin sensitivity check index (QUICKI) remained unchanged at both time-points, while C-peptide increased significantly by 39% (Δ: 0.69 ng/mL; *p* < 0.00001) and 51% (Δ: 0.91 ng/mL; *p* < 0.00001) from baseline at 1st and 2nd follow-ups, respectively (Table 1).

### Lipid compositions

**Figure 4:**
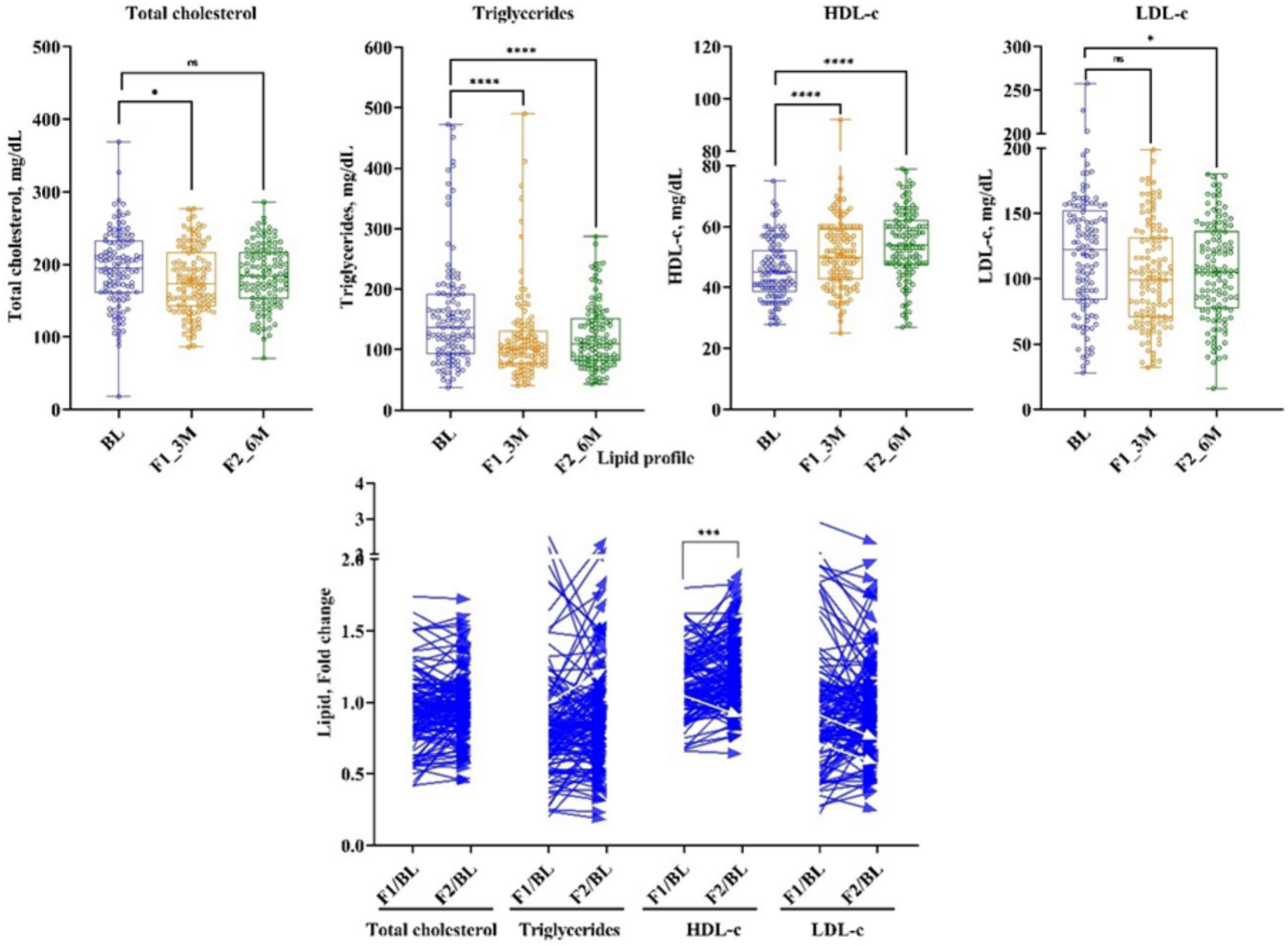
The effect of LCHF diet at 1st and 2nd follow-up on lipid profile. Baseline – BL; 1st follow-up at 3 months – F1_3M; and 2nd follow-up at 6 months – F2_6M. Data points for total cholesterol, triglycerides, HDL-c, and LDL-c presented in median (IQR). *N* = 119. *p*-value of <0.05 is considered significant. *p < 0.05, **p < 0.01, ***p < 0.001, and ****p < 0.0001.

Serum lipids reported a significant modulation after 3 and 6 months of the LCHF diet. (TG) reduced significantly by 22% (Δ: −27 mg/dL; *p* < 0.00001) and 14% (Δ: −19 mg/dL; *p* < 0.00001) at 1st and 2nd follow-ups, respectively, from baseline. Total cholesterol (TC) decreased by 5% (Δ: −10.5 mg/dL; *p* < 0.0436) at 1st follow-up, and these effects diminished at 2nd follow-up from baseline. Consequently, after 3 and 6 months of the LCHF diet, bad cholesterol, i.e., LDL-c has shown a significant reduction of 9.7% (Δ: −11.5 mg/dL; *p* < 0.02485) and 8% (Δ: −11 mg/dL; *p* < 0.02485), respectively, from baseline. Similarly, VLDL-c decreased at 1st and 2nd follow-up time-points by 21% (Δ: −5 mg/dL; *p* < 0.00001) and 14.5% (Δ: −4 mg/dL; *p* < 0.00001), respectively. Subsequently, good cholesterol, i.e., HDL-c increased by 11% (Δ: 5 mg/dL; *p* < 0.00001) and 17% (Δ: 8 mg/dL; *p* < 0.00001), respectively, from the baseline (Figure 4 and Table 1).

### Medication effect score (MES)

Diabetes medication use declined significantly during the 1st follow-up of the LCHF diet, and it remained unchanged at the 2nd follow-up from the baseline. MES decreased at 1st follow-up by 20% (Δ: −0.2; *p* < 0.02749). Nonetheless, at 2nd follow-up time-point, MES did not change from the baseline (Table 1).

### Concentration and correlation of tear’s secreted soluble protein and HbA1c

**Figure 5:**
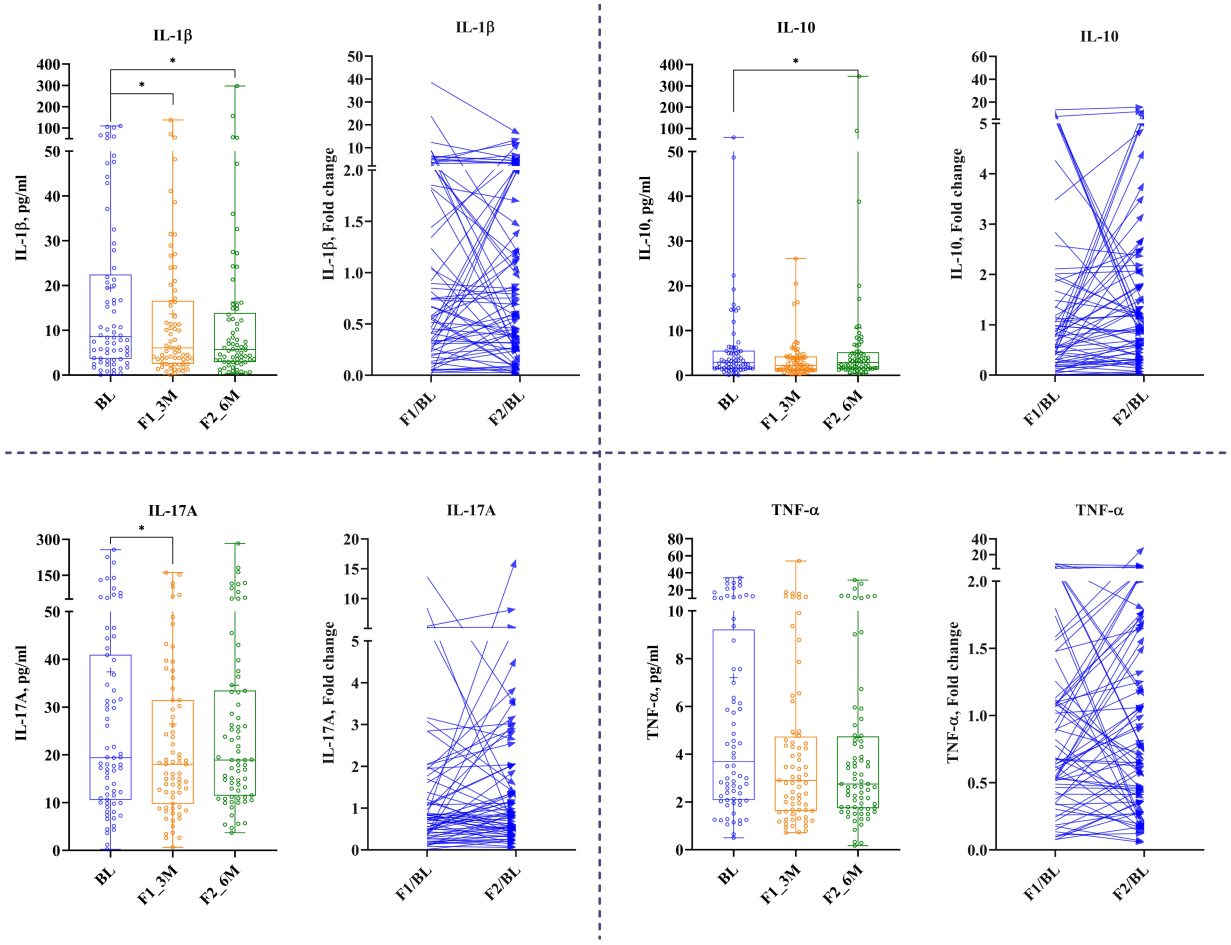
The effect of LCHF diet at 1st and 2nd follow-up on tear’s secreted soluble protein. Baseline – BL; 1st follow-up at 3 months – F1_3M; and 2nd follow-up at 6 months – F2_6M. Data points for IL-1β, ICAM-1, IL-17A, and TNF-α are presented in median (IQR). *N* = 119. *p*-value of <0.05 is considered significant. *p < 0.05, **p < 0.01, and ***p < 0.001.

Tear’s secreted soluble proteins such as IL-6, MMP-9, TNF-α, and VEGF-A remained unchanged at 1st follow-up, while the concentration of IL-10, IL-1β, and IL-17A reduced significantly by 19.5% (Δ: −0.42 mg/dL; *p* < 0.02671), 42% (Δ: −2.9 mg/dL; *p* < 0.02947), and 22% (Δ: −4.5 pg/mL; *p* < 0.02), respectively. At 2nd follow-up, IL-1β reduced significantly by 41% (Δ: −2.4 pg/mL; *p* < 0.02947) from the baseline; nonetheless, other analytes remained unchanged (Figure 5 and Table 2).

**Table 2:** Summary of tear and serum analytes at baseline and follow-up’s time-points^#^.

Variable	BL^1^			F1^2^			F2^3^			Friedman test (Paired sample)		
	*N*	Median	IQR	*N*	Median	IQR	*N*	Median	IQR	*N*	*p*-value^a^	Multiple comparisons
**Tear analytes (pg/mL)**												
IL_10	71	2.66	1.55–5.38	72	2.24	1.21–4.2	72	3.01	1.6–5.12	69	0.02671	(1) vs. (2)
IL_1β	72	8.87	3.78–21.6	71	5.96	2.82–16.34	73	5.79	3.21–14.83	70	0.02947	(1) vs. (2); (1) vs. (3)
IL_6	73	48.6	24.69–112.35	73	49.2	21.7–114	73	45.5	19.25–127.8	73	0.60553	–
MMP_9	72	74,826	26679.5–234,010	73	70,953	18771.75–189719.75	73	76,981	28912.75–200894.75	72	0.94667	–
ICAM_1	73	12087.5	7378.63–27218.25	73	10,631	6644.75–21437.5	73	11,361	6829.25–22565.25	73	0.18357	–
IL_17A	73	19.5	10.84–41.25	72	18.05	10.44–30.8	72	18.9	11.35–33.3	71	0.0386	(1) vs. (2)
TNF_α	73	3.86	2.11–8.91	73	2.91	1.66–4.64	72	2.75	1.76–4.68	72	0.0802	–
VEGF_A	73	1910.5	1261.38–3038.75	73	1,674	984.75–2859.75	73	1711	1142.5–2609.5	73	0.77324	–
**Serum analytes (pg/mL)**												
IL_10	57	4.34	2.07–6.34	57	3.75	2.31–5.59	57	3.65	2.34–5.48	57	0.23	–
IL_1β	8	5.53	0.75–10.26	11	2.03	0.95–4.29	5	0.87	0.64–5.6	2	–	–
IL_6	57	5.12	2.99–6.67	57	4.29	2.75–7.75	56	4.66	2.7–6.78	56	0.98261	–
MMP_9	57	168,813	134858.75–238284.75	57	212,002	156786.5–282198.5	57	172,226	135,186–275491.5	57	0.17365	–
ICAM_1	57	326,628	255,148–436593.75	57	322,566	240,267–381546.75	56	324,576	264699.5–444325.5	56	0.39866	–
IL_17A	15	5.39	2.65–16.23	8	3	2.34–4.6	4	6.36	3.65–9.92	0	–	–
TNF_α	57	32.4	22.65–36.73	57	28.2	21.95–34.18	56	28.1	22.7–34.7	56	0.10293	–
VEGF_A	57	360	216–670.25	57	319	194.75–501.75	55	365	235–754.75	55	0.28997	–

# All data are expressed as median (IQR). The *p*-value less than 0.05 considered significant, ^a^Friedman test (paired samples). ^1^BL – Baseline, ^2^F1 – 1st follow-up at 3 month from baseline, ^3^F2 – 2nd follow-up at 6 month from baseline; (1) vs. (2) – BL vs. F1; (1) vs. (3) – BL vs. F2; IL-1β (Interleukin 1 Beta), IL-6 (Interleukin 6), IL-10 (Interleukin 10), IL-17A (Interleukin 17A), MMP-9 (Matrix Metalloproteinase 9), ICAM-1 (Intercellular Adhesion Molecule 1), VEGF-A (Vascular endothelial growth factor A), and TNF-A (Tumor Necrosis Factor Alpha).

The correlation between HbA1c and tear’s secreted soluble protein is evaluated at the baseline and 1st and 2nd follow-up time-points, as shown in Table 3. While none of the tear’s analytes showed a correlation with HbA1c at either time-points, 1st fold change (1st follow-up/baseline) revealed that only IL-6 showed a weak negative correlation with HbA1c. Nonetheless, the correlation analysis between tear’s analytes and HbA1c showed no statistical significance at 2nd fold change (2nd follow-up/baseline). The levels of the various analytes measured were not significantly different between the right and left eyes of the subject at all the visits.

**Table 3:** Correlation of tear fluid secreted protein factors level with HbA1c at baseline and follow-up after LCHF diet intervention.

Tear analytes (pg/mL)										
	BL		F1		F2		F1/BL		F2/BL	
	*r*	*p*	*r*	*p*	*r*	*p*	*r*	*p*	*r*	*p*
IL-10	−0.0	0.983	−0.1	0.416	−0.0	0.985				
IL-1β	−0.14	0.256	−0.1	0.422	0.17	0.157				
IL-6	−0.03	0.819	−0.1	0.418	0.12	0.303	−0.24*	0.041	−0.08	0.501
MMP-9	−0.03	0.803	−0.07	0.579	0.12	0.322				
ICAM-1	−0.01	0.931	0.0	0.998	0.2	0.098			0.16	0.165
IL-17A	−0.1	0.405	0.09	0.475	0.04	0.728				
TNF-a	−0.19	0.114	0.01	0.922	0.08	0.531	−0.15	0.192	0.13	0.290
VEGF-A	0.15	0.196	−0.03	0.771	0.17	0.162			0.09	0.446
**Serum analytes (pg/mL)**										
IL-10	0.25	0.064	0.18	0.178	0.08	0.551	0.04	0.750	−0.04	0.779
IL-1β	−0.02	0.955	0.26	0.433			0.6	0.208	−1.0	
IL-6	0.04	0.756	0.25	0.065	0.12	0.396	−0.04	0.792	0.08	0.579
MMP-9	−0.11	0.399	0.14	0.285	0.01	0.949	−0.09	0.521	−0.09	0.485
ICAM-1	0.27*	0.039	0.03	0.842	0.06	0.637	0.09	0.485	0.07	0.624
IL-17A	−0.24	0.386	−0.69	0.060	−0.2	0.800	−1.0			
TNF-a	0.16	0.224	0.25	0.059	0.37**	0.005	0.02	0.901	0.17	0.223
VEGF-A	0.08	0.555	−0.05	0.704	0.15	0.279	0.14	0.296	0.17	0.222

BL – Baseline, F1 – 1st follow-up at 3 months from baseline, F2 – 2nd follow-up at 6 months from baseline, IL-1β (Interleukin 1 Beta), IL-6 (Interleukin 6), IL-10 (Interleukin 10), IL-17A (Interleukin 17A), MMP-9 (Matrix Metalloproteinase 9), ICAM-1 (Intercellular Adhesion Molecule 1), VEGF-A (Vascular endothelial growth factor A), and TNF-A (Tumor Necrosis Factor Alpha). *p < 0.05, **p < 0.01, ***p < 0.001, and ****p < 0.0001.

### Concentration and correlation of serum’s secreted soluble protein and HbA1c

At 1st and 2nd follow-ups, none of the serum analyte concentrations showed significant modulation from the baseline (Table 2).

The correlation between HbA1c and serum’s secreted soluble protein is evaluated at baseline and 1st and 2nd follow-up time-points, as shown in Table 3. At the baseline, only ICAM-1 showed a weak positive correlation with HbA1c; however, at 1st follow-up, none of the serum analytes showed any significant correlation with HbA1c. While at 2nd follow-up, TNF-α has shown a significant and moderately positive correlation with HbA1c. At 1st and 2nd fold changes, serum analytes showed no statistically significant correlation with HbA1c.

## Discussion

Hyperglycemia and T2DM have been extensively studied in the past, while a low- carbohydrate diet emerged as an attractive approach to prevent and reverse T2DM-associated health disorders. The results of the present study improve the understanding of the effects of the ketogenic diet/LCHF diet in subjects with T2DM for short- and long-term, more preciously in Indian subjects with T2DM. Consistent with the previous studies, in the South-Indian cohort, subjects with T2DM showed improvement in anthropogenic factors, such as weight, BMI, and WHR at the end of the study. Subsequently, there was an improvement in glycemic factors such as FBS, PPBS, and HbA1c, which corresponded to a reduction in dietary carbohydrate intake and elevation in fat intake. All these observations occurred without changing insulin and ketone concentration at the end of the study.

The possible reason for beneficial health delivery of the LCHF diet on subjects with T2DM could be restriction in the carbohydrate intake (Table 4), which leads to a decline in the absorption of sugar or monosaccharides lowering fasting and post-prandial blood glucose and regulating glucose and fatty acid metabolism (33, 34). The current study analyzed subjects at three different time-points, and the results showed a decrease in fasting blood sugar from diabetic range to pre-diabetic range. In addition, PPBS showed a drastic reduction at the end of the study, where approximately PPBS declined by 30%, indicating the effectiveness of the LCHF diet in lowering blood sugar.

**Table 4:** Macronutrient intake at baseline and follow-up by the participants and their changes after LCHF diet intervention^#^.

Variable	BL^1^			F1^2^			F2^3^			Friedman test (Paired sample)		
	*N*	Median	IQR	*N*	Median	IQR	*N*	Median	IQR	*N*	*p*-value^a^	Multiple comparisons
Carbohydrate, gm	42	322.9	302.4–341.3	42	115.3	85.0–146.3	42	125.5	95.0–228.9	42	<0.00001	(1) vs. (2); (1) vs. (3)
Fat, gm	42	48.3	42.1–56.0	42	130.6	121.6–141.4	42	125.9	83.3–134.2	42	<0.00001	(1) vs. (2); (1) vs. (3)
Protein, gm	42	55	49.0–60.0	42	59.5	51.0–63.0	42	58	55.0–62.0	42	<0.00001	(1) vs. (2); (1) vs. (3)

# Data presented in median (IQR); *p < 0.05, **p < 0.01, ***p < 0.001, and ****p < 0.0001, a Friedman test (paired samples). ^1^BL – Baseline, ^2^F1 – 1st follow-up at 3 months from baseline, ^3^F2 – 2nd follow-up at 6 months from baseline; (1) vs. (2) – BL vs. F1; (1) vs. (3) – BL vs. F2.

HbA1c was analyzed to evaluate the long-term effect of the LCHF diet. HbA1c level indicates crucial clinical significance about average plasma glucose of the previous 2–3 months for assessing the status of blood glucose control, which acts as a diagnostic tool for patients with diabetes and a screening test for persons at a high risk of diabetes progression (35). It has been reported that cardiac infractions and microvascular complications decreased by 14 and 37%, respectively, when HbA1c decreased by 1%. Thus, the HbA1c level established important clinical significance in estimating the blood glucose control, uncovering the potential challenges in the treatment and managing the therapeutic schedule (36, 37). In this study, a reduction in HbA1c occurred after 3 and 6 months of LCHF diet consumption; the changes appeared at −1% and −0.6% time-points; HbA1c reduced by 16 and 12.6%, respectively. The possible explanation for such small changes could be a wide range of ages; thus, the response of age to treatment cannot be neglected. The average cutoff of HbA1c was 1 in the current study, indicating that diabetes management may also be achieved by ketogenic diet/LCHF diet effects.

The ketogenic diet not only improved glucose metabolism, but several studies reported that the ketogenic diet enhanced lipid metabolism. For instance, Hussain et al. (38) showed that a ketogenic diet decreased TG and TC while increasing the HDL-c level significantly, and as a result, it recovers dyslipidemia-associated conditions. In the current study, at 1st and 2nd follow-ups, the LCHF diet showed that TG was reduced by 27 and 19 mg/dL, TC was reduced by 10.5 and 7 mg/dL, LDL-c was reduced by 11.5 and 11 mg/dL, while HDL-c was increased by 5 and 8 mg/dL, respectively, from the baseline. Similar results have been reported by Dashti et al. (39), where TG decreased by 3.67 mmol/L, TC decreased by 1.88 mmol/L, and LDL decreased by 1.78 mmol/L, while HDL increased by 0.14 mmol/L. Subsequently, the current study showed a reduction in systolic blood pressure by 7% (Δ: −10 mmHg; *p* < 0.0001) in subjects with T2DM. LCHF diet intake delivered significant positive effects in subjects with T2DM as elevated TG and free fatty acids (FFAs) are pathogenic factors for insulin resistance and oxidative stress and can lead to the escalation of risk for cardiovascular diseases (40, 41). Hence, recovery from dyslipidaemia is a positive outcome of the current study, which is not only beneficial for regulating insulin sensitivity but also for managing the incident and progression of diabetes-associated heart diseases (42, 43).

The diabetes medication was not restricted in the current study. While diabetes medication schedules are often complicated, with multiple agents, modified dosages, and frequent administration, it does not reduce the dependency on medication (29). There have been numerous studies that have explored reasons associated with non-adherence to anti-diabetic medication such as financial conditions, forgetfulness, younger age, education, ongoing diabetes complications, and problems in taking the medications alone (44, 45). Hence, diet could be a promising approach to maintaining health, reversing, and dependency on diabetes medication. In the current study, the LCHF diet shows a reduction in MES at 1st follow-up (*p* < 0.0001), which remained unchanged at 2nd follow-up from the baseline. However, this could be a possible lack of reporting by participants at 2nd follow-up time-point.

Diet has been proposed as one of the most commonly preferred modes to counter diabetes-associated disorders (46). Indian diet is rich in carbohydrates, ~60% (47), which is a high-carbohydrate diet, and plays a significant role in the development of insulin resistance and T2DM (48). Thus, a low-carbohydrate diet was focused on studying the effects of the LCHF diet on the reversibility of T2DM-associated clinical parameters, such as glycemic parameters, lipid profile, and medication score. To monitor the adherence of subjects to a low-carbohydrate diet, we examine macronutrients such as carbohydrates, fat, and protein at respective time-points. The results have shown that carbohydrate intake was reduced significantly from the baseline, while fatty acid intake elevated significantly (Table 4), and those changes were maintained at the end of the protocol. This revealed that subjects compiled the instruction of the diet throughout the study. To observe the effects of a low-carbohydrate diet at a systemic level, glycemic factors such as FBS, PPBS, HbA1c, and serum ketone were analyzed at respective time-points. At the end of the study, a low-carbohydrate diet reduced glycemic factors at the end of protocol, which supported the significance of a low-carbohydrate diet, consistent with the previous studies (39, 49). Similarly, the lipid profile was monitored to check the dyslipidaemia status of the subject during the study. Several studies have mentioned that dyslipidaemia plays a crucial role in the development of metabolic syndrome and T2DM (50). At the end of the study, total cholesterol, triglycerides, and LDL-c were reduced significantly while HDL-c decreased significantly. Medication is one of the effective ways to manage the consequences of T2DM. Nevertheless, as the disease progresses, uses, costs, and complications of glucose-lowering medication increased. For instance, more than 50% of patients with T2DM will require insulin within 10–15 years of diagnosis (51). Our current study revealed the significance of low-carbohydrate diet as it has reduced MES at respective time-points.

Tear fluid has gained prominence as a promising source for biomarker detection regarding the DR status due to its unique components, ease of collection, vicinity to the disease site, and minimal cell contamination (52, 53). Various systemic diseases have also been shown to reflect disease-associated alterations in the tear profiles. Several studies have demonstrated the presence of secreted soluble protein in tears, and alterations in tear quality and quantity have been reported in diabetic patients (54, 55). Furthermore, elevation in tear inflammatory factors has been revealed from various ocular diseases (4, 56). For instance, during inflammation, ICAM-1 expression is upregulated in the vascular endothelium of patients with DED (57). Similarly, a study showed that an elevation of protein and messenger RNA expression of ICAM-1 in lacrimal and conjunctival epithelial cells has been reported in patients with DED (58). In contrast, a study mentioned that diabetic mice deficient of ICAM-1 revealed a decrease in the preliminary lesions linked with DR (such as the damage of pericytes, degeneration, and increased capillary permeability) and leukostasis (59). Moreover, the expression of TNF-α has been associated with the pathogenesis of various chronic inflammatory disorders, such as T2DM (60). Diabetes patients have shown a higher concentration of TNF-α in plasma than non-diabetic, where a strong correlation was found between TNF- α and severity of DR (61). Similarly, TNF-α was found higher in diabetic patient’s tears while similar results were observed in serum and vitreous (62). A study revealed that the TNF-α concentration in tears escalates with the severity of DR, the concentration being less in non-diabetic participants than in diabetic, and correspondingly, the correlation of TNF-α was found highly significant with DR severity (63). Interestingly, the intravitreal injection of TNF-α inhibitor resulted in lowering of damage of pericytes and capillary degeneration in diabetic mice (64, 65). Similarly, TNF-α-deficient mice demonstrated a reduction in vascular changes induced by diabetes (66). In summary, soluble secreted proteins of tears such as inhibition of ICAM-1 and TNF-α exert valuable effects on the prevention of early diabetic retinopathy (59, 67). Overall, data propose that inflammation and ocular diabetic complications are interconnected, and that, the concentration of the circulating inflammatory factor may estimate the stages and development of DR. As the tear sample collection technique is non-invasive for patients and easy for technicians (68), so far in Indian population with diabetes, there have been no reports related to the alteration in secreted soluble protein in tears of diabetic subjects treated with low-carbohydrate diet. Hence, in the present study, we demonstrated the outcomes of analysis of soluble secreted protein (IL-1b, IL-6, IL-8, IL-10, IL-17A, MMP-9, ICAM-1, VEGF-A, and TNF- α) in tears, which was collected from Indian subjects with T2DM. At 1st follow-up, IL-10, IL-1β, and IL-17A were decreased significantly, while IL-1β was decreased significantly at 2nd follow-up. In contrast to previous observation, the present study revealed that there was no significant recovery of diabetes corresponding to TNF-α and ICAM-1 in serum and tears. However, a large set of data is needed to verify the outcomes and prediction of biomarkers in tears, indicating the status of DR after diet intervention. Spearman’s correlation analysis was performed on HbA1c to evaluate relationship strength with tear analytes, but none of the correlations were statistically significant (Table 3).

Tear protein levels have been associated with a variety of ocular conditions and systemic diseases. In our subject cohort, we aimed to distinguish if specific molecular factors correlated with the HbA1c variability could be used as a monitoring tool to examine T2DM progression. Of course, further studies with a larger number of participants and longer duration are conducted to test the idea of whether tear analytes have a predictive function in the appearance of hyperglycemia. One of the limitations of our study is that more participants are recruited to include further subject stratification into sub-groups. Furthermore, our data illustrate another limitation of the current study that some of the study subjects likely already had insulin resistance, as demonstrated by the data analysis in the results. By having a separate insulin-sensitive or naïve diabetic group, we could have obtained information on the difference between treated and non-treated and T2DM and non-T2DM and their correlation with analytes. Finally, the study cohort lacks advanced diabetic retinopathy subjects, which are difficult to include due to ongoing laser or intravitreal treatments. More studies are required to examine the early changes with possible predictive value, leading to reversibility of hyperglycemia and insulin resistance, and the results should be validated on independent cohorts. It is also important to remark that the current study worked well for dyslipidemia and dysglycemia but did not show significant differences for a set of tear analytes.

Despite the above-mentioned limitations, our study reveals information that can be used as potential biomarkers for monitoring studies aiming at personalizing therapies and offering alternative solutions for insulin resistance monitored in advanced obesity and T2DM.

## Data availability statement

The raw data supporting the conclusions of this article will be made available by the authors, without undue reservation. The clinical data can be shared after de-identification due to ethical clearance.

## Ethics statement

The studies involving humans were approved by the Ethics Committee of Narayana Nethralaya (EC reference No: NNIEC/2022/02/01). The studies were conducted in accordance with the local legislation and institutional requirements. The participants provided their written informed consent to participate in this study. Written informed consent was obtained from the individual(s) for the publication of any potentially identifiable images or data included in this article.

## Author contributions

NB: Data curation, Formal analysis, Methodology, Software, Visualization, Writing – original draft, Investigation, Writing – review & editing. KBS: Investigation, Methodology, Resources, Writing – original draft. NS: Writing – review & editing, Investigation, Methodology, Resources. KS: Data curation, Writing – review & editing, Investigation, Methodology. AK: Data curation, Writing – review & editing, Investigation, Methodology. NP: Funding acquisition, Resources, Writing – review & editing, Methodology, Project administration. RS: Supervision, Writing – review & editing, Conceptualization, Funding acquisition, Resources. AG: Project administration, Supervision, Validation, Writing – review & editing, Conceptualization, Data curation, Formal analysis, Funding acquisition, Methodology.

## In memoriam

The authors thank late Dr. Bhujang K. Shetty at the Narayana Nethralaya Eye Hospital, Bangalore, for his initiative and invaluable assistance in organizing and performing this study.
